# Supplementary material for: TERRA recruitment of polycomb to telomeres is essential for histone trymethylation marks at telomeric heterochromatin
Source: Nat Commun. 2018 Apr 18;9:1548. doi: 10.1038/s41467-018-03916-3 (PMC5906467; doi:10.1038/s41467-018-03916-3)
Supplement: Supplementary file 1 — Supplementary Information [file 41467_2018_3916_MOESM1_ESM.pdf]

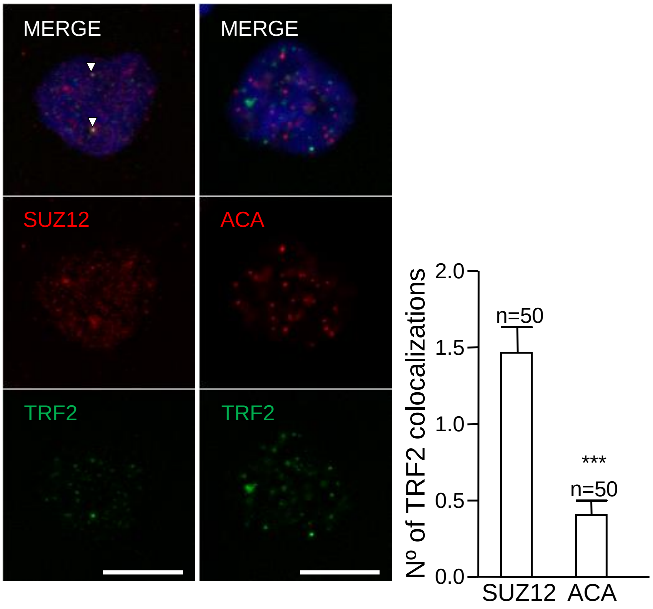

Supplementary Figure 1. Quantification of the random colocalization between the centromere and the telomeric protein TRF2. Representative images of the average number of colocalizations found on double inmunostaining to detect the telomere protein TRF2 (green) and the PRC2 protein SUZ12 (red) (Left panel) or between TRF2 (green) and the centromere using the Anti-Centromere Antibody (ACA) (red) (Right panel) in U2OS cells. (*Graph*) Quantification of the co-localization between TRF2 and SUZ12 or TRF2 and ACA. Arrowheads indicate co-localization events. Scale bar, 10 *µm*. Student's t-test was used for the statistical analysis (*p <0.05, ** p <0.01 and *** p <0.001).

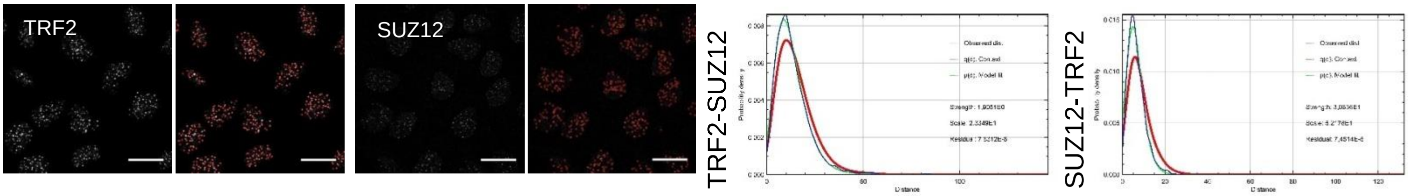

Supplementary Figure 2. MosaicIA radomization approach to prove the interaction of SUZ12 with the telomere. Representative images of object identification using MosaicIA interaction plugin of Fiji for TRF2 and SUZ12 signal. Red circles: Identified objects. Scale: 20*µM*. (*Graphs*) Graphical representation of the comparison of the interacting potential between SUZ12 and TRF2. Blue line: distribution of NND between objects identified in TRF2 images vs SUZ12 images. Green line: modelization of interaction potential using a Plummer potential. Red line: probability density function resulting of the calculation of NND between TRF2 and SUZ12 images if the objects in TRF2 were distributed randomly, in a completely independent manner of the distribution of SUZ12 (l*eft graph*). Similarly, it was also calculated for SUZ12 objects distributed randomly, in a completely independent manner of the distribution of TRF2 (*right graph*). Strength is a measure of the degree of dependence between TRF2 and SUZ12 objects distribution. The strength is superior to zero, indicating that the spatial distribution of TRF2 is dependent upon the spatial distribution of SUZ12. When compared against 10000 Monte Carlo samples of point distributions corresponding to the null hypothesis of “no interaction”, the results are statistically significant (*p*<0,001).

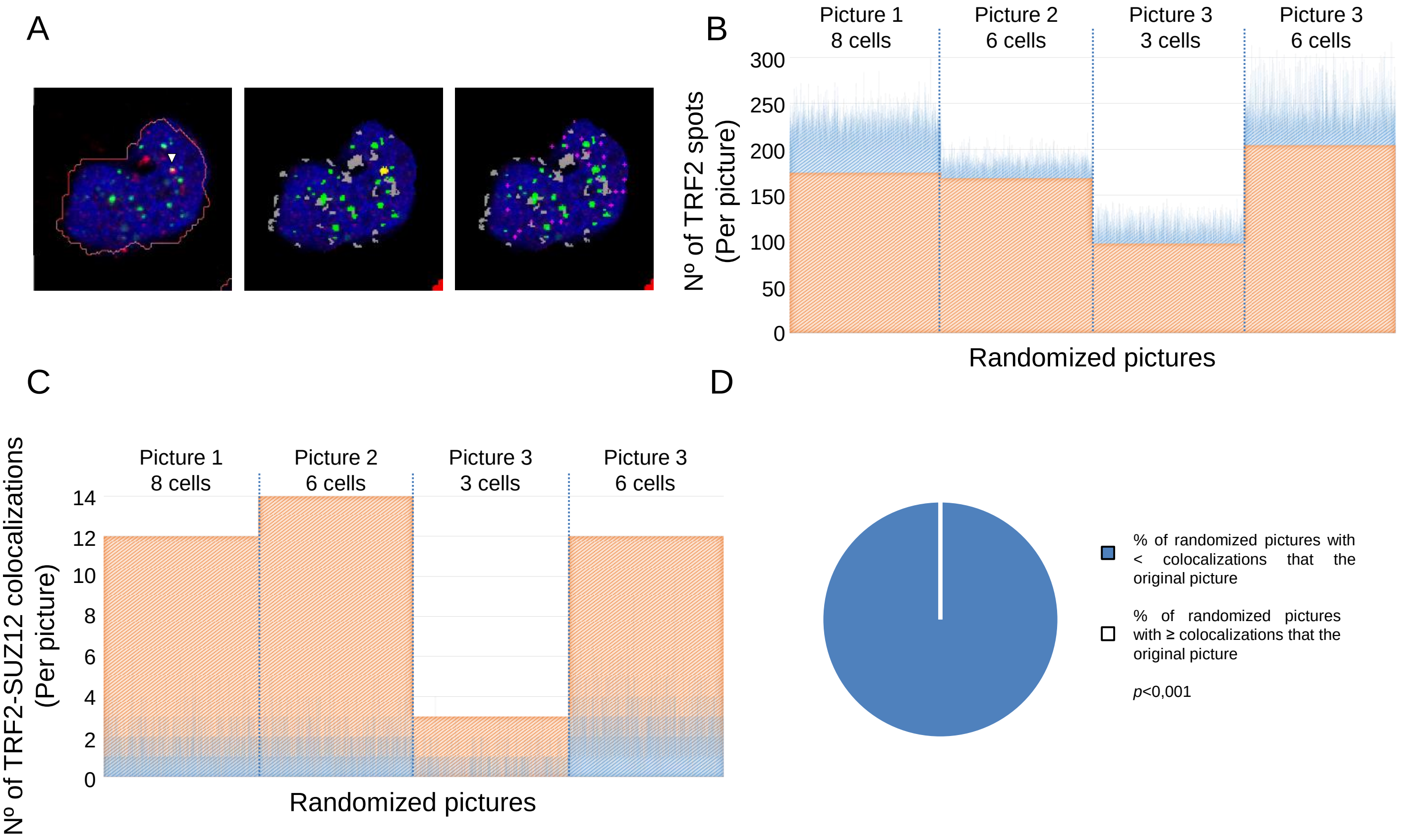

Supplementary Figure 3. Definiens radomization approach to prove the interaction of SUZ12 with the telomere. (A) Representative images of the co-localizations found on double inmunostaining to detect the telomere protein TRF2 (green) and the PRC2 protein SUZ12 (red). (*Left*) Nuclear identification by the Definiens Developer XD.2 software(red line). Arrowheads indicate real co-localization events. (*Middle*) Representative example of the TRF2 spots identification by the Definiens Developer XD.2 program (green). Co-localization events identified by the program is shown in yellow. The gray areas represent areas of low DNA signal that were not used for the analysis. (*Right*) Representative example of a randomized image generated by the Definiens Developer XD.2 program. Virtual TRF2 spots are shown (magenta). **(B)** Graphical representation of the total real and virtual TRF2 spots identified by the Definiens Developer XD.2 program in four different pictures (each picture contains different number of nuclei). The orange bars represents the number of real TRF2 spots in the originals pictures and the blue bars represent the virtual TRF2 spots generated by the program in every random permutation. **(C)** Graphical representation of the total real and virtual TRF2-SUZ12 co-localizations events identified by the Definiens Developer XD.2 program in four different pictures. The orange bars represents the number of TRF2-SUZ12 real co-localizations identified in the originals pictures and the blue bars represent the virtual TRF2-SUZ12 co-localizations identified in every random permutation. **(D)** Diagram showing the % of randomized pictures with < co-localizations that the original picture versus the % of randomized pictures with ≥ co-localizations that the original picture. The result show that the co-localization events between SUZ12 and TRF2 are not due to random colocalization and this is statistically significant (p<0,001). The p-value is calculated as the probability of finding a randomized picture with ≥ co-localizations than in the original picture. This is calculated with the number of randomized pictures with ≥ co-localizations than in the original picture divided by the total number of randomized pictures.

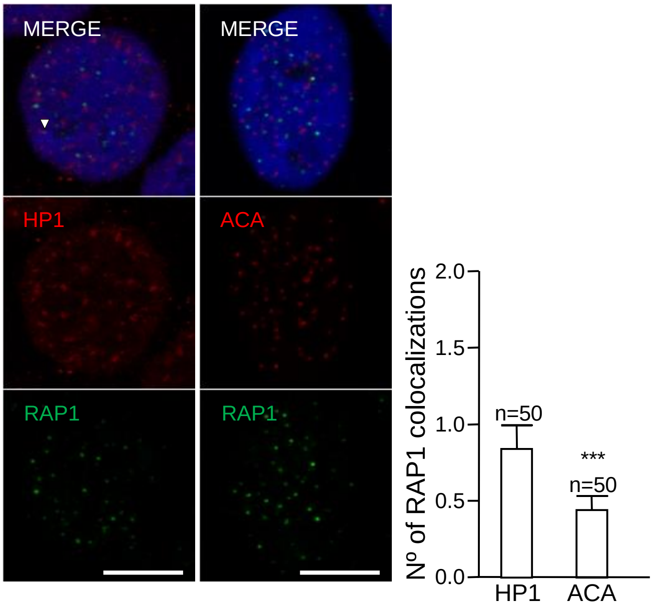

Supplementary Figure 4. Quantification of the random colocalization between the centromere and the telomeric protein RAP1. Representative images of the average number of colocalizations found on double inmunostaining to detect the telomere protein RAP1 (green) and the HP1 protein (red) (Left panel) or between RAP1 (green) and (ACA) (red) (Right panel) in U2OS cells. (*Graph*) Quantification of the co-localization between RAP1 and HP1 or RAP1 and ACA. Arrowheads indicate co-localization events. Scale bar, 10 *μm*. Student's t-test was used for the statistical analysis (*p <0.05, ** p <0.01 and *** p <0.001).

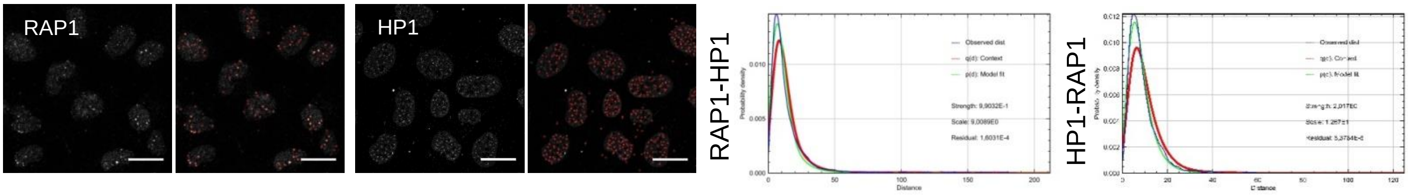

Supplementary Figure 5. MosaicIA radomization approach to prove the interaction of HP1 with the telomere. Representative images of object identification using MosaicIA interaction plugin of Fiji for RAP1 and HP1 signal. Red circles: Identified objects. Scale: 20*µM*. (*Graphs*) Graphical representation of the comparison of the interacting potential between HP1 and RAP1. Blue line: distribution of NND between objects identified in RAP1 images vs HP1 images. Green line: modelization of interaction potential using a Plummer potential. Red line: probability density function resulting of the calculation of NND between RAP1 and HP1 images if the objects in RAP1 were distributed randomly, in a completely independent manner of the distribution of HP1(l*eft graph*). Similarly, it was also calculated for HP1 objects distributed randomly, in a completely independent manner of the distribution of RAP1. Strength is a measure of the degree of dependence between RAP1 and HP1 objects distribution. The strength is superior to zero, indicating that the spatial distribution of RAP1 is dependent upon the spatial distribution of RAP1. When compared against 10000 Monte Carlo samples of point distributions corresponding to the null hypothesis of “no interaction”, the results showed are statistically significant (p<0,001).

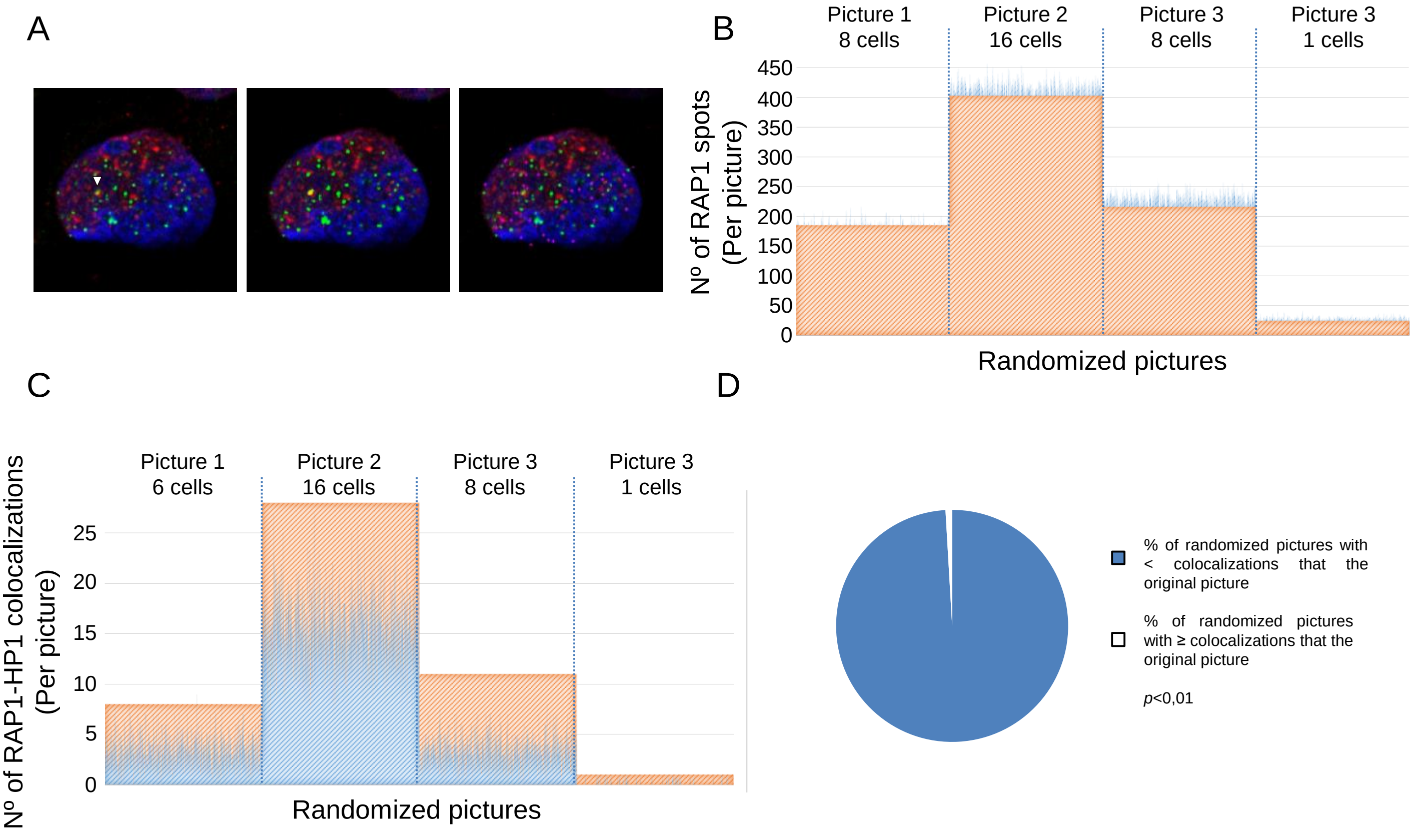

Supplementary Figure 6. Definiens radomization approach to prove the interaction of HP1 with the telomere. (A) Representative images of the co-localizations found on double inmunostaining to detect the telomere protein RAP1 (green) and the HP1 protein (red). (*Left*). Arrowheads indicate real co-localization events. (*Middle*) Representative example of the RAP1 spots identification by the Definiens Developer XD.2 program (green). Co-localization events identified by the program is shown in yellow. (*Right*) Representative example of a randomized image generated by the Definiens Developer XD.2 program. Virtual RAP1 spots are shown (magenta). (**B**) Graphical representation of the total real and virtual RAP1 spots identified by the Definiens Developer XD.2 program in four different pictures (each picture contains different number of nuclei). The orange bars represents the number of real RAP1 spots in the originals pictures and the blue bars represent the virtual RAP1 spots generated by the program in every random permutation. **(C)** Graphical representation of the total real and virtual RAP1-HP1 co-localizations events identified by the Definiens Developer XD.2 program in four different pictures. The orange bars represents the number of RAP1-HP1 real co-localizations identified in the originals pictures and the blue bars represent the virtual RAP1-HP1 co-localizations identified in every random permutation. **(D)** Diagram showing the % of randomized pictures with < co-localizations that the original picture versus the % of randomized pictures with ≥ co-localizations that the original picture. The result show that the co-localization events between HP1 and RAP1 are not due to random colocalization and this is statistically significant (p<0,01). The p-value is calculated as the probability of finding a randomized picture with ≥ co-localizations than in the original picture. This is calculated with the number of randomized pictures with ≥ co-localizations than in the original picture divided by the total number of randomized pictures.

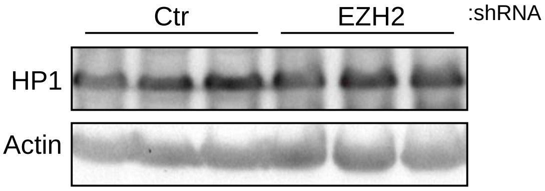

Supplementary Figure 7. HP1 levels do not change upon EZH2 donwregulation. U2OS cells were infected with a lentivirus encoding a shRNA against EZH2. After puromycin selection, total protein extracts were obtained and use for western blot detection of HP1. Actin was used as loading control.

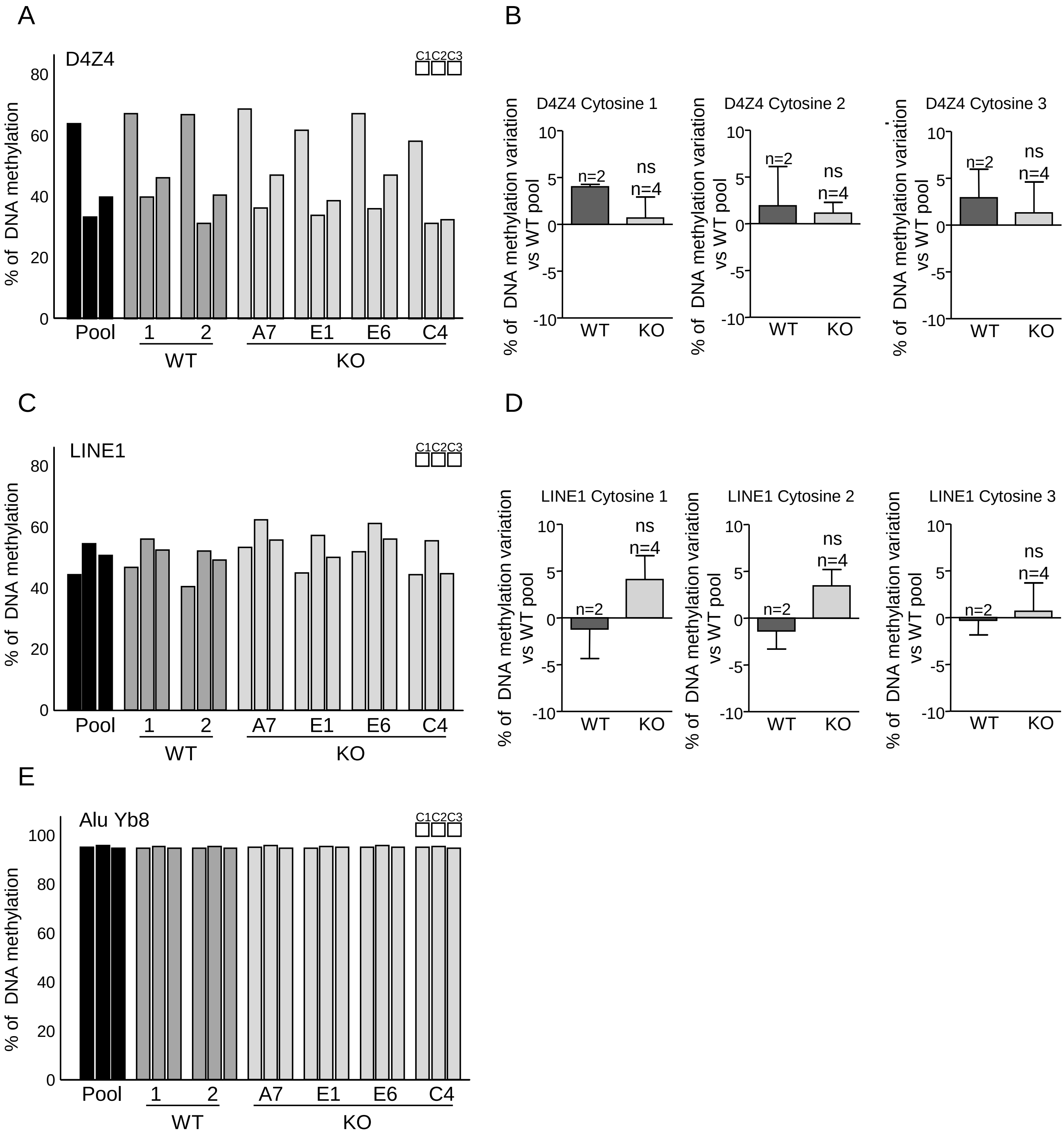
A
D4Z4
C1C2C3
% of DNA methylation
80
60
40
20
0
Pool
1
2
A7
E1
E6
C4
WT
KO
B
D4Z4 Cytosine 1
D4Z4 Cytosine 2
D4Z4 Cytosine 3
% of DNA methylation variation vs WT pool
10
5
0
-5
-10
n=2
ns
n=4
WT
KO
C
LINE1
LINE1 Cytosine 1
LINE1 Cytosine 2
LINE1 Cytosine 3
D
E
Alu Yb8
100

Supplementary Figure 8. TERRAs do not change subtelomeric and global DNA methylation. (A) Graph showing the percentage of methylation of the subtelomeric repeat D4Z4 by bisulfite pyrosequencing at the first three cytosines (C1, C2 and C3) in the U2OS WT pool, WT clones (#1 and 2) and in the 20q-TERRA KO clones (#A7, E1, E6, and C4). **(B)** Percentage of DNA methylation variation in the subtelomeric repeat D4Z4 respect to the WT pool in the WT clones and in the 20q-TERRA KO (mean values±s.e.m., n=independent clones). **(C)** Graph showing the percentage of DNA methylation of the Line1 repeats by bisulfite pyrosequencing at the first three cytosines in the U2OS WT pool, WT clones (#1 and 2) and in the 20q-TERRA KO clones (#A7, E1, E6, and C4). **(D)** Percentage of DNA methylation variation in the Line1 repeat respect to the WT pool in the WT clones and 20q-TERRA KO clones (mean values±s.e.m., n=independent clones). **(E)** Percentage of methylation of Alu repeat by bisulfite pyrosequencing at the first threet cytosines in the U2OS WT pool, WT clones (#1 and 2) and in the 20q-TERRA KO clones (#A7, E1, E6, and C4). The methylation at Alu repeats are use as control. Student's t-test was used for the statistical analysis (*p<0.05, ** p <0.01 and *** p <0.001).

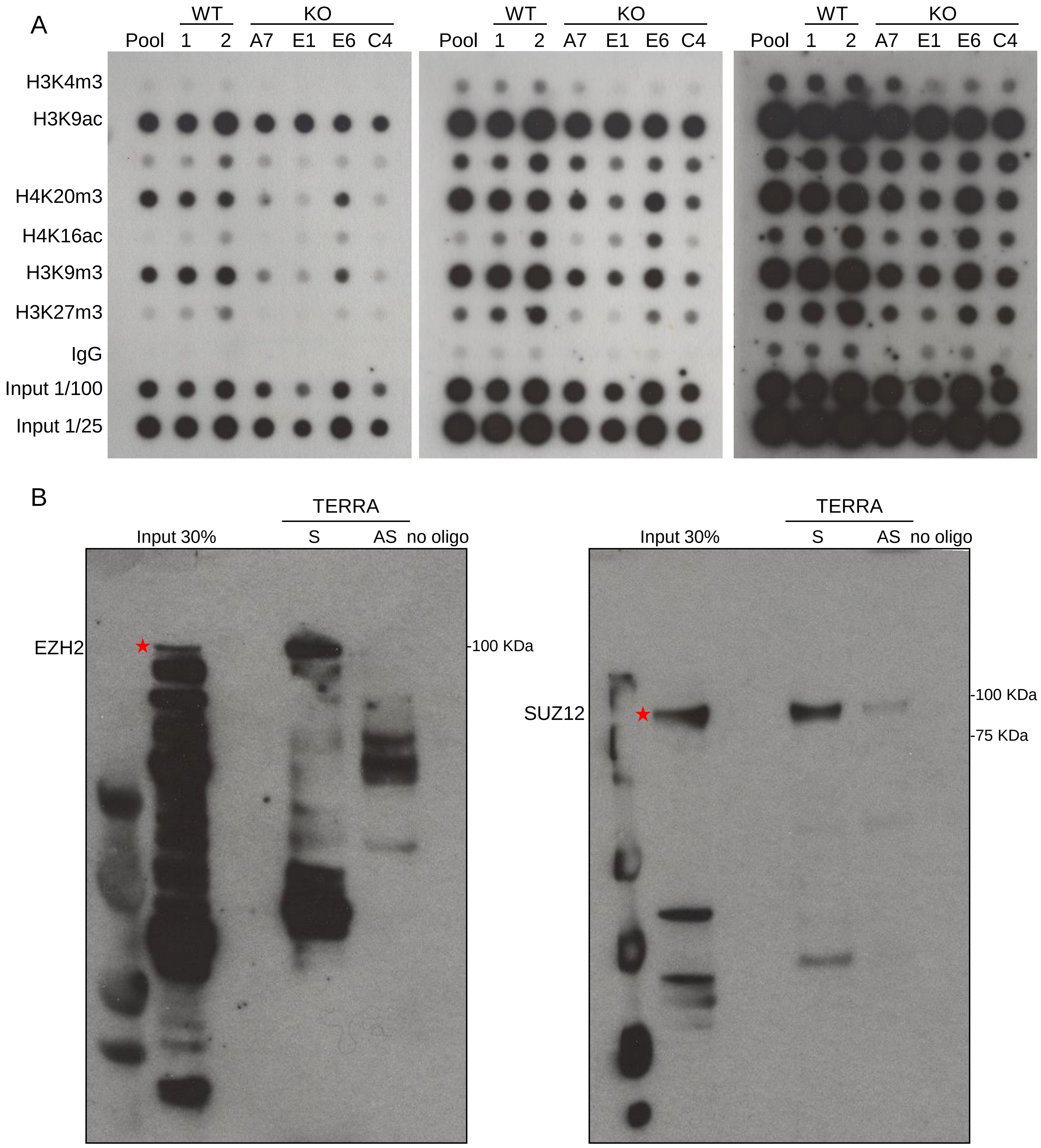

A
WT
KO
Pool
1
2
A7
E1
E6
C4
H3K4m3
H3K9ac
H4K20m3
H4K16ac
H3K9m3
H3K27m3
IgG
Input 1/100
Input 1/25
B
TERRA
Input 30%
S
AS
no oligo
EZH2
100 KDa
SUZ12
100 KDa
75 KDa

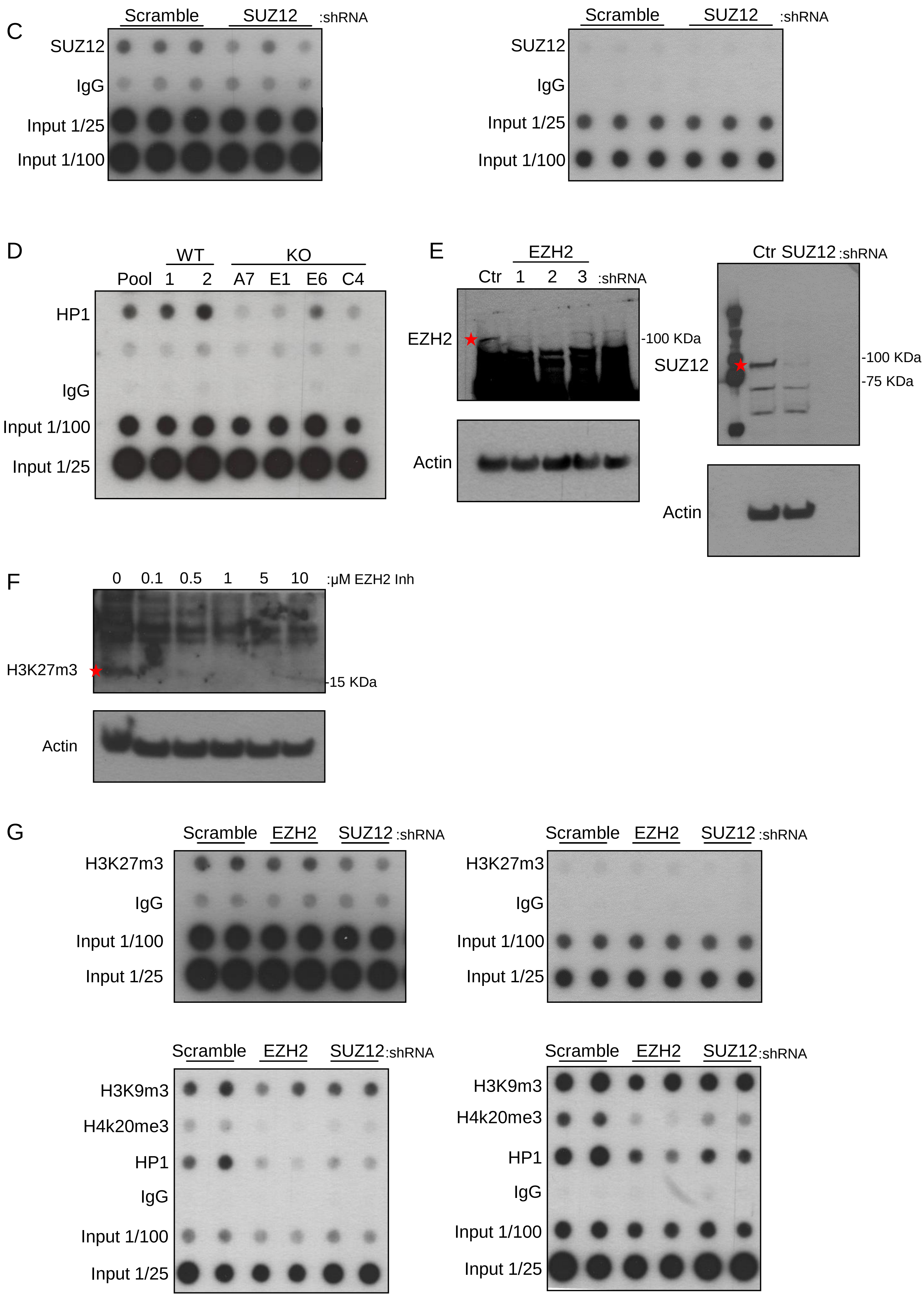
C
Scramble
SUZ12
:shRNA
SUZ12
IgG
Input 1/25
Input 1/100
Scramble
SUZ12
:shRNA
SUZ12
IgG
Input 1/25
Input 1/100
D
WT
KO
Pool
1
2
A7
E1
E6
C4
HP1
IgG
Input 1/100
Input 1/25
E
EZH2
Ctr
1
2
3
:shRNA
EZH2
-100 KDa
Actin
Ctr SUZ12 :shRNA
SUZ12
-100 KDa
-75 KDa
Actin
F
0
0.1
0.5
1
5
10
:μM EZH2 Inh
H3K27m3
-15 KDa
Actin
G
Scramble
EZH2
SUZ12 :shRNA
H3K27m3
IgG
Input 1/100
Input 1/25
Scramble
EZH2
SUZ12 :shRNA
H3K27m3
IgG
Input 1/100
Input 1/25
Scramble
EZH2
SUZ12:shRNA
H3K9m3
H4k20me3
HP1
IgG
Input 1/100
Input 1/25
Scramble
EZH2
SUZ12:shRNA
H3K9m3
H4k20me3
HP1
IgG
Input 1/100
Input 1/25

Supplementary Figure 9. Full Blots and Gels. (A) Full ChIP Dot-Blot from the main figure 3. Different exposures are shown. Only relevant lanes are labeled. **(B)** Full Western Blot from the main figure 4A. **(C)** Full ChIP Dot-Blot from the main figure 4C. Different exposures are shown. **(D)** Full ChIP Dot-Blot from the main figure 4E. Only relevant lanes are labeled. **(E)** Full Western Blot from the main figure 5A. **(F)** Full Western Blot from the main figure 5B. **(G)** Full ChIP Dot-Blot from the main figure 5D and 5F. Different exposures are shown. In the Western Blots red stars indicated the bands shown in the main figure.

Supplementary Table 1. shRNAs and primers

.

Primer	Sequence (5´-3´)
shRNA scramble	CCTAAGGTTAAGTCGCCCTCGCTCGAGCGAGGGCGACTTAACCTTAGG
shRNA EZH2 1	GCTAGGTTAATTGGGACCAAACTCGAGTTTGGTCCCAATTAACCTAG
shRNA EZH2 2	CCAACACAAGTCATCCCATTACTCGAGTAATGGGATGACTTGTGTTGG
shRNA EZH2 3	CCCAACATAGATGGACCAAATCTCGAGATTTGGTCCATCTATGTTGGG
shRNA SUZ12	GCTGACAATCAAATGAATCATCTCGAGATGATTCATTTGATTGTCAGC
F-U620qE1Not	GCGAGcggccgcacatgtgagggcctatttcc
R-U620qE1Not	CGGTGcggccgcAtggggagagtgaagcagaa
